# Large Sigmoid Fecaloma: A Rare Case of a Common Condition in Patients With Parkinson’s Disease

**DOI:** 10.7759/cureus.44523

**Published:** 2023-09-01

**Authors:** Resheed Alkhiari

**Affiliations:** 1 Department of Medicine, College of Medicine, Qassim University, Qassim, SAU

**Keywords:** colonoscopy, parkinson's disease, fecaloma, fecal impaction, chronic constipation

## Abstract

A fecaloma is a rare complication of chronic constipation that is more commonly seen in elderly individuals with chronic neuropsychiatric disorders. We present the case of a 79-year-old patient with Parkinson’s disease with refractory constipation due to a large fecaloma mass in the sigmoid colon, which is a rare sequela of poorly managed chronic constipation. The current report highlights the importance of aggressive medical therapy for chronic constipation in this group of patients to prevent life-threatening complications.

## Introduction

A fecaloma is a solid fecal mass that usually forms over time due to stasis of fecal matter secondary to chronic constipation [1]. There are many reports of fecalomas in patients with colonic dysmotility related to diseases involving colonic innervation such as Chagas and Hirschsprung’s diseases. The major pathophysiology of fecalomas involves untreated constipation, which leads to fecal impaction and fecaloma formation [2]. Fecalomas are associated with a considerable risk of life-threatening conditions, including large bowel obstruction, colonic perforation, and very rarely life-threatening lower GI hemorrhage. The standard treatment is endoscopic or surgical removal of the fecaloma [3-5]. We present a case of this rare condition and discuss endoscopic therapy, which is a minimally invasive therapy for high-risk populations.

## Case presentation

**Figure 1:**
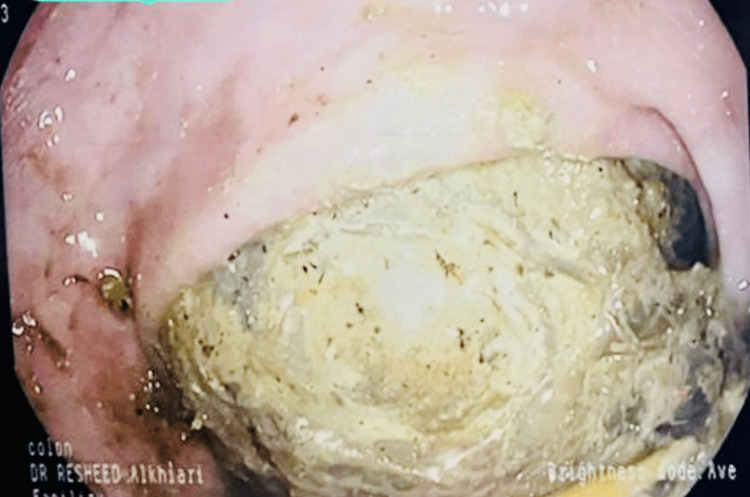
Colonoscopy view of a large fecaloma in the sigmoid colon

A 79-year-old female was referred to our clinic with chronic constipation and left lower abdominal pain persisting for four months. The constipation was progressive and resistant to multiple osmotic and stimulant laxatives. Her abdominal pain was described as intermittent and colicky and occurred mainly around the time of passing a bowel movement. She denied any rectal bleeding but reported a weight loss of five kilograms over two months. She had multiple medical problems, including diabetes mellitus for two years on metformin, hypertension, coronary artery disease, and Parkinson’s disease. Her Parkinson's disease was diagnosed seven years ago and she had been stable on levodopa and carbidopa with mild tremors and stiffness with no limitations to her basic daily activity. She reported a history of chronic constipation for more than four years and had never undergone a colonoscopy. She has tried multiple laxatives, including fibers and Senna in addition to daily polyethylene glycol. On examination, she was independent in most of her daily activities and used a cane for walking. Her vital signs were within normal limits, and her body mass index was 28 kg/m^2^. Abdominal examination showed a distended abdomen with mild lower abdominal tenderness and no palpable masses or organomegaly. Her recent blood work showed a normal calcium level, normal thyroid function testing, and normal hemoglobin. A colonoscopy procedure was then arranged to evaluate her refractory constipation. During the colonoscopy, we identified a large semi-obstructing fecaloma in the sigmoid colon (Figure 1); otherwise, the colonic mucosa was normal. Endoscopic removal was performed successfully using regular endoscopic forceps and a snare combined with water irrigation to fragment the fecaloma. The patient was then placed on regular polyethylene glycol to treat her constipation and was seen for follow-up in the clinic for two months. She experienced complete resolution of her abdominal symptoms and significant improvement in her constipation.

## Discussion

Chronic constipation is a chronic, common condition that occurs in approximately 8.2% of the general population; however, the prevalence in elderly individuals is as high as 18% due to many factors, including limited mobility, reduced fluid intake or limited fluid intake due to cardiac or renal impairment, and polypharmacy [6]. If constipation is neglected or inadequately treated, fecal impaction or fecaloma formation can occur and lead to unfavorable outcomes [7].

Parkinson's disease is a motor neurodegenerative disease that has a progressive nature and leads to autonomic dysfunction in end-stage disease. It mainly affects the elderly with an incidence of 1% after the age of 60 [8]. The prevalence of constipation in Parkinson's disease is high, up to 80%, due to many factors including impaired smooth muscles of the intestine related to autonomic dysfunction, limited mobility, and reduced intestine secretions as a side effect of medications used to control Parkinson's disease [9].

A fecaloma is a rare complication that was first reported in 1967 [1]. This condition can mimic malignancy on abdominal imaging. Fecalomas are commonly located in the left colon, particularly in the rectum and sigmoid; in very rare cases, they have been reported in the small intestine. The majority of reported cases have linked fecalomas to motility disorders associated with chronic constipation and neuropsychiatric conditions as well as to any narrowing of the colonic lumen related to inflammatory, neoplastic, or post-surgical anastomosis [2,5].

The literature suggests that there are many complications associated with fecalomas, including stool incontinence due to overflow diarrhea, urine retention, and chronic abdominal pain secondary to compression of adjacent structures [10,11]. Other serious complications include bowel obstruction, ulcer formation, fistula, perforation, massive lower gastrointestinal bleeding, and death. Mortality in patients with fecalomas is reportedly associated with old age, the presence of perforation, and co-existing comorbidities such as chronic renal failure and neuropsychiatric disorders [12].

The standard treatment for fecalomas can be divided into three strategies: conservative treatment and endoscopic or surgical removal [5,4,12]. Conservative therapy consists of manual fragmentation, rectal enema administration, and oral laxative (polyethylene glycol) administration [5,13]. Endoscopic therapy is usually considered when conservative therapy is unsuccessful; this treatment includes colonoscopy and direct water irrigation to dissolve the fecaloma assisted by different endoscopic tools, including needles, snares, and forceps, to fragment the fecaloma to aid in removal using endoscopic suction [4]. A surgical approach is usually the last resort but can be the first-line treatment when a patient has experienced perforation or unhealed ulceration [10,12].

## Conclusions

This case report highlights a very rare complication of common chronic constipation in a patient with Parkinson’s disease as well as the need for careful evaluation when constipation is refractory to medical therapy. Fecaloma formation can be prevented with extra care for chronic constipation by providing balanced water intake, fiber, and laxatives.
